# Academic Impact of Hand Surgery Units Across the United Kingdom: A Bibliometric Analysis

**DOI:** 10.7759/cureus.27782

**Published:** 2022-08-08

**Authors:** Norbert F Banhidy, Francis P Banhidy

**Affiliations:** 1 Plastic Surgery, Royal London Hospital, London, GBR; 2 Plastic and Reconstructive Surgery, University College London, London, GBR

**Keywords:** orthopaedic hand surgery, plastic and reconstructive surgery, academic research, bibliometric analyis, h index

## Abstract

Quantifying the academic impact of hand surgery units can serve as a useful parameter for clinicians interested in academia when applying for fellowships or consultant posts. The aim of this study is to measure and rank the academic impact of hand surgery units across the United Kingdom (UK) using bibliometric analysis. UK hand surgery units were identified from the British Society for Surgery of the Hand (BSSH) website and additional manual internet searches. Predefined search strings were used to identify papers about or relating to hand surgery. Using the Clarivate Analytics Web of Science bibliometric analysis tool, cumulative (1900-2021), 10-year (2011-2021), and 3-year (2018-2021) research output data was collected from UK hand surgery units and ranked using the following parameters: number of papers (Np), number of citations (Nc), and the h-index (a metric evaluating the cumulative impact of academic output). The top three units according to the 10-year h-index were The Pulvertaft Hand Centre (15), John Radcliffe Hospital (10), and Norfolk and Norwich University Hospital (10). The units with the greatest number of papers published in the last 10 years were the Pulvertaft Hand Centre (70), Chelsea & Westminster Hospitals (45), and Broomfield Hospital (44). The units with the single most cited papers were Wrightington Hospital (189), the Pulvertaft Hand Centre (152), and St John’s Hospital & Royal Hospital for Sick Children (152). The academic impact of hand surgery units varies greatly across the UK. Hand surgery units with a historically strong academic record have generally maintained a similar high output of research over the last decade. The 10-year h-index of hand surgery units can be particularly useful for hand surgeons with a strong academic interest.

## Introduction and background

Evaluating the academic impact of individual clinicians and institutions by means of bibliometric analysis has gained significant interest in the last decade [1]. The academic impact of clinical departments greatly influences decision-making in research fund allocation by health funding organizations both in the United Kingdom (UK) and internationally [2]. Furthermore, the academic output of individual clinicians is a strong predictor of promotion and career progression in academic surgery [3].

Historically, academic output measurements have relied solely on productivity metrics such as the number of papers published (Np) and author status (first author, second author,... last author) [4]. These parameters demonstrate the academic throughput of researchers over time. However, they do not shed light on the scholarly influence of the individual or the research impact that each paper generates. In contrast, impact metrics such as the number of citations (Nc) and impact factor (IF) consider the overall academic influence of the individual paper and publishing journal, respectively [4].

The Hirsch index (h-index) is a metric that amalgamates the productivity and impact profiles of Np and Nc, respectively. Developed by Hirsch in 2005, the h-index provides "an estimate of the importance, significance, and broad impact of a scientist’s cumulative research contributions" [5]. The h-index is calculated by identifying the maximum number of papers that have gained the maximum number of citations. To give an example, a department with an h-index of 9 would mean that at least nine papers have been cited nine times originating from that department. The h-index has greater predictive power than Np, Nc, or IF in determining future academic success [6], making it exceedingly relevant for employment and promotion decisions, as well as guiding research funding decisions [7,8].

The h-index has been used in the field of surgery to evaluate individual and departmental scholarly output across a number of specialties [9,10]. A significant proportion of bibliometric data on academic surgery originates from the United States (US), but more recently in the UK, academic output comparisons have been published in areas such as neurosurgery and plastic surgery [11,12]. However, no such comparison exists across hand surgery departments. Hand surgeons with an interest in academia may benefit from comparing hand surgery units based on academic output when choosing a department for employment or when considering research funding applications. As such, this study aims to use the h-index, amongst other bibliometric parameters, to compare the relative academic output of hand surgery units across the UK.

## Review

Materials and methods

A list of hand surgery units across the United Kingdom was obtained from the British Society for Surgery of the Hand (BSSH) website. The historical names of individual hospitals were identified through manual searches to capture all data from departments whose names had changed or merged with other hospitals. Hand surgery was defined per the BSSH as the "assessment and management of conditions affecting the hand, wrist, and peripheral nerves of the upper limb" [13]. The following search strings were produced with the aid of a librarian search strategist to identify relevant articles: (hand OR finger* OR nail bed OR digit* OR wrist OR thumb* OR phalan* OR matacarpal OR carp* OR scapho* OR lunate OR capitate OR hamat* OR trapez* OR CMCJ OR Carpometacarpal* OR Dupuytren* OR De Quervain* OR Boutonniere*). The Clarivate Analytics Web of Science was used on September 30, 2021, to obtain bibliometric data using the search strings combined with the name of the respective unit in the "address field" of the search criteria. All articles were screened independently by both authors to ensure relevance. Bibliometric data on Np, Nc, and the h-index were collected for each hand surgery unit over three distinct time periods, including cumulative data since records began (1950-2021), 10-year data from 2011 to 2021, and 3-year data from 2018 to 2021.

Results

A total of 68 hand surgery units were identified in the UK. Of these, 61 had produced research articles that were identifiable in the database search. A total of 1405 articles were identified in the cumulative time period spanning from 1950 to 2021.

The bibliometric data on all units for the cumulative, previous 10-year and 3-year periods are shown in Table 1. The units are ranked according to the 10-year h-index. The top 3 units according to the 10-year h-index were the Pulvertaft Hand Centre (15), John Radcliffe Hospital (10), and Norfolk and Norwich University Hospital (10). The units with the greatest Nc over a 10-year period were, similarly, the Pulvertaft Centre (785), John Radcliffe Hospital (291), and Norfolk and Norwich University Hospital (274). The greatest Np over the last 10 years came from the Pulvertaft Hand Centre (70), Chelsea & Westminster Hospital (45), and Broomfield Hospital (44).

**Table 1:** All UK hand surgery units ranked according to 10-year h-index [h(10)] The table includes h-index (h), number of papers published (Np) and number of citations (Nc) for three time periods: cumulative (1950-2021), 10-year (2011-2021) and 3-year (2018-2021). The table also shows the Nc for the highest cited paper from each unit.

h(10) Rank	Unit	Cumulative			10-year			3-year			Top paper Nc total
		Np	Nc	h	Np	Nc	h	Np	Nc	h	
1	Pulvertaft Hand Centre, Royal Derby Hospital	136	2254	27	70	785	15	28	25	2	182
2	John Radcliffe Hospital	42	365	10	28	291	10	14	46	4	118
2	Norfolk and Norwich University Hospital	51	66	16	33	274	10	8	6	2	71
4	Nottingham City Hospital	30	370	10	23	191	9	11	29	4	73
5	Broomfield Hospital	82	819	16	44	237	8	19	39	3	94
6	Wrightington Hospital	58	1178	17	24	127	6	12	19	3	196
6	Queen Victoria Hospital	97	771	15	42	129	6	18	9	2	105
6	Glasgow Royal Infirmary	41	226	9	26	116	6	16	45	3	43
6	Leeds General Infirmary	47	165	7	38	130	6	15	26	2	28
10	Charing Cross & St Mary’s Hospital	45	273	9	23	69	5	12	12	2	68
10	Addenbrooke’s Hospital	76	981	19	23	94	5	11	28	3	65
10	Royal United Hospital	17	157	6	11	93	5	5	2	1	57
10	Aberdeen Royal Infirmary	26	228	8	8	67	5	0	0	0	50
10	Chelsea & Westminster Hospital	58	228	7	45	108	5	29	43	3	42
10	Ninewells Hospital	23	105	6	17	72	5	4	12	2	34
10	James Cook University Hospital	17	147	7	9	100	5	2	0	0	34
17	The Royal Free Hospital	47	340	10	32	69	4	19	26	2	104
17	Derriford Hospital	16	184	6	10	67	4	1	0	0	85
17	Great Ormond Street Hospital	18	113	7	11	42	4	2	9	1	20
17	Whiston Hospital	16	61	4	7	41	4	4	26	2	16
21	Salford Royal Hospital	8	161	4	4	28	3	3	17	2	113
21	Wexham Park Hospital	32	247	8	10	24	3	2	1	1	73
21	Castle Hill Hospital	19	149	6	8	97	3	3	2	1	68
21	Morriston Hospital	31	209	8	9	15	3	4	3	1	67
21	The Royal London Hospital	15	97	5	10	18	3	9	11	2	40
21	Birmingham Children’s Hospital	19	65	4	14	27	3	4	3	1	30
21	Alder Hey Children’s Hospital	15	103	6	7	22	3	2	0	0	30
21	Queen Alexandra Hospital	11	47	4	8	23	3	3	0	0	20
21	Royal National Orthopaedic Hospital	4	31	3	4	31	3	3	21	3	13
21	Queen Elizabeth Hospital & Selly Oak	21	47	4	19	40	3	7	17	2	13
21	Southmead Hospital	7	34	3	7	34	3	5	16	2	13
32	St John’s Hospital & Royal Hospital for Sick Children	25	307	8	10	13	2	2	1	1	152
32	Leicester Royal Infirmary	36	883	15	5	7	2	2	0	0	119
32	Royal Victoria Infirmary	18	271	6	5	12	2	4	6	1	73
32	Bradford Royal Infirmary	9	52	3	3	6	2	2	3	1	28
32	Stoke Mandeville Hospital	20	74	5	8	11	2	5	11	2	22
32	Countess of Chester Hospital	13	62	5	7	20	2	4	1	1	16
32	University Hospital of Coventry and Warkwickshire	8	28	2	8	28	2	6	17	2	13
32	Royal Sussex County Hospital	5	7	2	3	5	2	0	0	0	2
40	Wythenshawe Hospital	13	425	20	1	24	1	0	0	0	83
40	Guy’s & St Thomas' Hospital	24	338	11	3	4	1	2	4	1	67
40	The Lister Hospital	11	84	5	2	1	1	1	0	0	41
40	Pinderfields Hospital	28	91	5	11	3	1	3	2	1	38
40	Kettering General Hospital	4	53	3	2	13	1	1	1	1	31
40	Salisbury District Hospital	8	87	5	2	7	1	0	0	0	30
40	St George's Hospital	5	34	2	3	11	1	1	0	0	23
40	Trafford General Hospital	2	11	2	1	2	1	1	2	1	9
40	Birmingham City Hospital & Sandwell Hospital	12	16	2	6	9	1	2	0	0	9
40	Forth Valley Royal Hospital	1	7	1	1	7	1	1	7	1	7
40	Royal Stoke University Hospital	2	3	1	2	3	1	1	3	1	3
40	Wirral University Teaching Hospital	2	2	1	1	2	1	0	0	0	2
52	Royal Marsden Hospital	1	135	1	0	0	0	0	0	0	135
52	Royal Hallamshire Hospital	9	149	7	0	0	0	0	0	0	47
52	University Hospital of North Durham	3	42	2	0	0	0	0	0	0	24
52	Royal Devon and Exeter Hospital	7	29	2	1	0	0	0	0	0	23
52	York Hospital	2	20	2	0	0	0	0	0	0	17
52	Wansbeck General Hospital	2	21	2	0	0	0	0	0	0	15
52	Royal Preston Hospital	6	29	4	1	0	0	0	0	0	11
52	Northampton General Hospital	2	7	1	1	0	0	0	0	0	7
52	West Suffolk Hospital	1	3	1	0	0	0	0	0	0	3
52	Russells Hall Hospital	1	1	1	0	0	0	0	0	0	1
52	Royal Bournemouth Hospital	0	0	0	0	0	0	0	0	0	0
52	Royal Victoria Hospital	0	0	0	0	0	0	0	0	0	0
52	Freeman Hospital	0	0	0	0	0	0	0	0	0	0
52	Bristol Royal Hospital	0	0	0	0	0	0	0	0	0	0
52	Southampton University Hospital	0	0	0	0	0	0	0	0	0	0
52	The Christie	0	0	0	0	0	0	0	0	0	0
52	Royal Orthopaedic Hospital	0	0	0	0	0	0	0	0	0	0

The top five papers with the highest number of citations over a cumulative time period are presented in Table 2. The top five cited papers originate from Wrightington Hospital, Pulvertaft Hand Centre, St John’s Hospital & Royal Hospital for Sick Children, Royal Marsden Hospital, and Leicester Royal Infirmary, respectively. The top five papers were published between 1985 and 2006.

**Table 2:** Top five papers published according to number of citations in the UK by hand surgery units

Rank	Unit	Paper	Journal	Year	Nc
1	Wrightington Hospital	Three-ligament tenodesis for the treatment of scapholunate dissociation: Indications and surgical technique	Journal of Hand Surgery - American Volume	2006	196
2	Pulvertaft Hand Centre, Royal Derby Hospital	Excision of the trapezium for osteoarthritis of the trapeziometacarpal joint: A study of the benefit of ligament reconstruction or tendon interposition	Journal of Hand Surgery - American Volume	2004	182
3	St John’s Hospital & Royal Hospital for Sick Children, Edinburgh	The distally-based dorsal hand flap	British Journal of Plastic Surgery (Continued as Journal of Plastic, Reconstructive and Aesthetic Surgery)	1990	152
4	Royal Marsden Hospital, London	Acute-ischemia of the hand resulting from elevation of a radial forearm flap	British Journal of Plastic Surgery (Continued as Journal of Plastic, Reconstructive and Aesthetic Surgery)	1985	135
5	Leicester Royal Infirmary	Need the thumb be immobilised in scaphoid fractures - a randomized prospective trial	The Journal of bone and joint surgery - British volume (Continued as Bone and Joint Journal)	1991	119

Discussion

Hand surgery has historically held a very strong academic profile. Many key studies stand out as having had a long-lasting and far-reaching impact on the field, such as the advances in the diagnosis and treatment of carpal tunnel syndrome by Phalen [14]; Bunnel’s work on tendon repairs [15]; and the first reported digital artery repair by Kleinert [16], to only name a few. Despite this, many important questions still remain unanswered by hand surgeons, even for more common hand disorders, such as the optimum management of distal radius fractures [17].

The vast majority of high-impact articles originate from the US, with the UK and other countries trailing considerably behind. As illustrated by a bibliometric study published in 2013, 76 of the top 100 cited papers in hand surgery came from the US, with only six being attributed to the UK [18]. This is in keeping with the literature reporting on the wider academic dominance of the US in other surgical specialties [19]. 

Steps are being made to address both the gaps in knowledge in hand surgery and the UK’s role in contributing to this research. The James Lind Alliance, a UK-based non-profit organisation, together with the BSSH, curated a list of research priorities concerning common hand and wrist conditions [20]. This priority-setting partnership allowed the identification of the research questions at hand, which, when answered, would yield the greatest impact for patients and health care providers alike.

This is the first study to therefore evaluate the academic impact of hand surgery units across the UK. The data in Table 1 demonstrate that certain units, such as the Pulvertaft Hand Centre, John Radcliffe Hospital, and Norfolk and Norwich University Hospital have been able to maintain their relatively high academic impact throughout the last 70 years. Meanwhile, the academic impact of other historically well-performing units, including Leicester Royal Infirmary and Wythenshawe Hospital, has dropped in more recent years. Finally, units including Royal National Orthopaedic Hospital and Chelsea & Westminster Hospital have ascended the rankings of academic impact over time. The reason for these trends is not completely explained by the data and it is likely multifactorial, but the data clearly demonstrate a strong correlation between h-index, Np, and Nc. This finding is consistent with studies of academic impact in other surgical specialties [11,12], and suggests that the quality and quantity of research produced by units are closely connected.

Examining the top five most cited papers in Table 2 also reveals some noteworthy trends. All the top-cited papers were published between 1985 and 2006. Citations tend to increase with time, so older articles will naturally have been cited more times on average than recent articles. This observation does not, however, account for the comparative lack of highly cited papers before 1980. This "golden age" of articles with high citation rates between the 1980s and early 2000s is also replicated in a bibliometric study examining global hand surgery research [18], and the wider field of plastic surgery [12]. This relative spike in citations may well be driven by the increasing emphasis on evidence-based medicine at the time [21], accompanied by a magnified focus on academia and the increased use of metrics such as the impact factor [22].

The findings presented in this study have multiple useful implications. First, the study reinforces that the h-index is an easily measurable and comparable metric, which may be applied to both individual researchers or whole departments across a broad range of specialties. The 10-year h-index is a particularly effective measure of current academic research output. Second, the results may prove beneficial to both current academic hand surgeons and prospective clinicians aiming to join a department. Regular review and audit of a department’s academic output through its h-index may be used to ensure continued high quality and quantity of research as well as a benchmarking metric for research grant applications. Prospective academic surgeons may use the data to guide career decisions and to facilitate their advancement in the field of academic hand surgery.

Despite the study’s usefulness, it is not without its limitations. The scope of the database search is inherently limited by the number of articles available on the Web of Science database. As the Web of Science lists most but not all journals, this may have a small effect on the overall data retrieved. It is unlikely, however, that this would have a significant effect on the study findings, as most high-impact hand surgery articles appear in common journals indexed by the Web of Science.

Although the search strategy employed by the authors ensured that relevant articles were included, it is possible that the predefined search strings restricted the search and excluded a subset of otherwise relevant articles. This potential limitation was mitigated by enlisting the help of a librarian to improve and validate the authors’ search strings. Furthermore, due to the constantly changing nature of hand surgery and broader health care provision in the UK, several departments have changed names, merged or closed over the years. Efforts were made to include all historic names and account for changes in hand surgery department status by conducting manual searches individually by both authors. However, it is possible that certain names were not captured. It is also worth noting that the publications of individual high-output academic surgeons may affect the overall academic impact of their units, especially when working in smaller units. However, as this study aimed to assess the academic impact of hand surgery units as a whole, it does not provide a breakdown of individual authors.

Finally, it is important to note that the Web of Science makes no distinction between articles based on their levels of evidence. As such, all articles, ranging from case reports to randomised control trials and systematic reviews, have been analysed as a single unstratified pool of articles. Conclusions on the robustness of evidence and the methodology of hand surgery units may therefore not be drawn from the study findings.

## Conclusions

This is the first study to compare the academic output of hand surgery units across the UK using bibliometric analysis. The findings demonstrate that the academic impact of hand surgery units varies greatly across the UK. Hand surgery units with a historically strong academic record have generally maintained a similar high output of research over the last decade. The findings validate the use of the 10-year h-index as a quick and easy measure of current departmental academic output and may be used as a research benchmarking tool and indicator of future scholarly output. The methodology may be easily applied to other specialties to produce useful bibliometric data. As such, further studies should focus on regular academic output assessments of a broad range of specialties as well as stratifying the levels of evidence presented in the published articles.
